# 

**Published:** 1967-12

**Authors:** 


BRISTOL MEDICO-CHIRURGICAL JOURNAL

*(Incorporating the Medical Journal of the South-West)*

Established 1 8 8 3

Vol. 82 (iv) December, 1967 No. 306

### *Contents*

| | Page |
|---|---|
| THE COLLECTING OF WILD PLANTS with special reference to those of Medicinal or Toxicological Importance ... *T. F. Hewer* | 97 |
| A DISTRICT NURSE/GP ATTACHMENT ... *W. B. Fletcher* | 107 |

*PUBLISHED QUARTERLY*

*ANNUAL SUBSCRIPTION* **16/-** *POST FREE*

### BRISTOL MEDICO-CHIRURGICAL SOCIETY

President 1966-67 : Dr. S. F. Marwood.

President-elect : Professor T. F. Hewer.

Honorary Secretary : Dr. A. T. M. Roberts, 8 Harley Place, Bristol 8.

Honorary Treasurer : Dr. J. Macrae, Ham Green Hospital, Pill.

The Society was founded in 1874. In 1883 publication of this Journal began, and has continued quarterly since then. During the years 1949 to 1962 it appeared under the title of "The Medical Journal of the South West".

The Society founded a medical library in 1890, which in 1892 was combined with that of the Bristol Medical School. This became the University of Bristol Medical Library in 1925, and an agreement in that year confirmed the privilege of Members to use the University Medical Library.

### THE BRISTOL MEDICO-CHIRURGICAL JOURNAL

*Editorial Committee*

Dr. N. J. Brown, Southmead Hospital, Bristol. *Joint Editor.*

Dr. A. B. Raper, Department of Pathology, Bristol Royal Infirmary. *Joint Editor.*

Dr. J. Macrae, Ham Green Hospital, Pill, Bristol. *Honorary Treasurer.*

Dr. R. C. Wofinden, Central Clinic, Bristol.

Mr. A. E. S. Roberts, Medical School Library, University Walk, Bristol 8. *Secretary.*

### NOTICE TO CONTRIBUTORS

The Journal is especially concerned to provide a place for the recording of medical thought, interests, and practice in and around Bristol. It therefore welcomes articles that, as well as being of immediate interest to members, will document the local and contemporary medical scene for our successors.

Original articles are invited provided that they have not been published elsewhere. References in the text should be by author's name, with year of publication in parenthesis; they should be listed at the end in alphabetical order. Illustrations should be supplied ready for reproduction. Line drawings should be in indian ink on firm white paper or board. The author will be informed if it is found necessary to ask him to pay for the making of blocks. The Editoral Committee reserves the right to make literary corrections.

The following contributions will be especially welcome: notes of forthcoming events, meetings held, degrees conferred, staff appointments, honours and public appointments and other local news.

### NOTES AND NEWS

Dr. John Apley has succeeded D. Philip Evans as leader of the British paediatric team in Saigon. His assignment has been organised by the Ministry of Overseas Development under the Colombo Plan technical assistance arrangements, and he will be away for one year.

Mr. H. G. Dixon, F.R.C.O.G., has been appointed to the chair of Obstetrics and Gynacology at the University of Bristol.

Dr. D. S. Ferguson has been awarded a U.S. Public Health Service fellowship for 1967-8.

Mr. R. E. Horton has become a member of the Court of Examiners of the Royal College of Surgeons.

---

### EXAMINATION RESULTS

M.R.C.P. London—

Dr. R. C. Wofinden

M.D. Bristol—

Bimla Arora
June Muriel Brown
Philip Frederick Harris
Edward Michael Sedgwick
Ivor Surveyor

Bristol—

Ronald Arthur Sellwood

Ph.D., Faculty of Medicine, Bristol—

Leon Martin Silverstone

D.P.H. Bristol—

M. K. E. Allington

D. H. Forster

S. F. Husseini

S. Z. Khanam

R. E. Midwinter (with Distinction)

V. Pansini

M. Postiglione

M. Rahman

N. Rifai

R. M. Robertson

J. S. Rodgers

W. H. Wernsdorfer

M.B., Ch.B., Bristol—

E. J. Leese, with Second Class Honours, and Distinction in Public Health
B. R. A. Maddox, with Second Class Honours

I. M. Vickery, with Second Class Honours
G. B. Salmon, with Distinction in Public Health

C. Adey

G. A. J. Appleby

A. F. Avery

A. A. J. Barros d'Sa

R. E. Barry

A. F. Boyles

B. J. Burke

P. E. Caville

S. K. M. Chan

M. C. Clark

P. B. Clark

A. G. Clarke

D. F. Cochrane

Y. F. Dajani

M. I. Delves

D. W. Felse

S. Gage

D. W. R. Gibson

T. J. Hamblin

W. A. V. Henderson

V. M. Hesketh

J. D. L. Holroyd

M. S. Jones

S. E. Mooney

W. J. Moore

J. F. Morris

H. G. Oon

D. Poole

M. B. Roberts

M. J. Y. Ruck

R. J. Simpson

D. J. T. Webster

### BRISTOL MEDICO-CHIRURGICAL SOCIETY

### MEETINGS HELD, 1966-1967

12th October, 1966 — Presidential Address, Dr. S. F. Marwood.

9th November, 1966 — Clinical Meeting, Southmead Hospital

14th December, 1966 — "General Practitioner Obstetrics—the Ten Commandments", Mr. John Crossley.

11th January, 1967 — "The Nature of Man", the Rt. Rev. Mervyn Stockwood.

8th February, 1967 — "Rheumatoid Arthritis—An Historical Perspective", Dr. John A. Cosh.

8th March, 1967 — Symposium, "The Intensive Care Unit", Miss G. M. Westbrook, Dr. Gordon Mather, Mr. Gerald Keen.

12th April, 1967 — "Is your Handicap really necessary?", Prof. Neville Butler.

10th May, 1967 — "Pain, Time, and Awareness", Dr. David Stafford-Clark.

